# Adherence to DASH-Style Dietary Pattern Impacts on Adiponectin and Clustered Metabolic Risk in Older Women

**DOI:** 10.3390/nu11040805

**Published:** 2019-04-09

**Authors:** Andreas Nilsson, Patrik Halvardsson, Fawzi Kadi

**Affiliations:** School of Health Sciences, Örebro University, 70182 Örebro, Sweden; pathah132@studentmail.oru.se (P.H.); fawzi.kadi@oru.se (F.K.)

**Keywords:** diet, dietary inflammatory index, inflammation, CRP, moderate-to-vigorous physical activity, aging

## Abstract

While dietary patterns are related to the age-related progression of chronic diseases, to what extent different dietary patterns influence inflammatory and metabolic risk factors in older adults remains to be elucidated. Additionally, potential moderating effects by physical activity (PA) become important to clarify. Here, we hypothesize that dietary patterns are linked to inflammatory and metabolic biomarkers and that these links are independent of PA. The present study aims to explore links between two dietary constructs and biomarkers of systemic inflammation and metabolic health in older women, while considering time spent in moderate-to-vigorous PA (MVPA). A cross-sectional analysis of a sample of 112 community-dwelling older women (65–70 years old) was performed. Dietary constructs based on the Dietary Approach to Stop Hypertension (DASH) and the dietary inflammatory index (DII) were determined from food records. MVPA was objectively assessed using accelerometry. Metabolic outcomes (waist circumference, systolic/diastolic blood pressures and levels of glucose, triglycerides and high-density lipoprotein (HDL)-cholesterol) and inflammatory biomarkers (C-reactive protein (CRP), fibrinogen and adiponectin) were determined using standardized procedures and a clustered metabolic risk score was derived. Adherence to DASH-style diet was significantly (*p* < 0.05) associated with a lower clustered metabolic risk, where women in the highest adherence group had a significantly (*p* < 0.05) lower waist circumference and blood glucose level compared to those in the lowest group. Further, a significantly higher (*p* < 0.05) adiponectin level was observed in the high DASH adherence group compared to those with low adherence. Notably, adjustment by waist circumference did not alter links with either adiponectin or blood glucose level. Importantly, all observed links remained significant after further adjustment for time in MVPA. Finally, no significant associations were observed when the dietary pattern was defined by the DII. The findings of this study demonstrate that DASH-style diets promote a systemic anti-inflammatory environment, while also mitigating clustered metabolic risk in older women. A key finding is that favourable impacts of the DASH-style diet are independent of time spent in moderate-to-vigorous PA, which further strengthens healthy eating behaviours as a key target for clinical and public health interventions designed to prevent age-related metabolic abnormalities.

## 1. Introduction

Cardiovascular disease (CVD) and type 2 diabetes represent chronic diseases with major impacts on the global health burden [1]. Although the pathophysiological mechanisms underlying disease progressions are not fully understood, a cluster of metabolic abnormalities (abdominal obesity, hypertension, dyslipidemia and hyperglycemia) has been related to the development of both CVD and type 2 diabetes [2]. Additionally, an increasing body of evidence points to the important role played by chronic systemic inflammation in the progression of cardiometabolic disorders [3]. Therefore, combating the progression of metabolic risk factors and the related systemic inflammation is essential to reduce chronic disease risk. Accordingly, dietary and physical activity habits have been identified as key modifiable lifestyle behaviours with the potential to readily impact on metabolic risk factors and systemic inflammation [4,5,6,7].

While health-related effects of single nutrients on disease risk have commonly been determined in previous research, attention has grown towards the impact of overall dietary quality on health outcomes. Indeed, dietary qualities have been linked to reduced risk of metabolic abnormalities, including lower clustered metabolic risk [8,9], which supports the whole-diet health strategy currently endorsed by global health authorities [10]. For the purpose of assessing dietary quality, several constructs considering different aspects of dietary intake have been developed. The Dietary Approach to Stop Hypertension (DASH) is a construct recommended by the American Heart Association and is based on food items/groups related to increased or decreased cardiovascular risk (e.g., fruits and vegetables, red meat and sodium) [11]. Another construct, the dietary inflammatory index (DII), has been developed to evaluate the inflammatory load of the diet on the basis of a literature review that examined the evidence between dietary intake and biomarkers of inflammation [12]. While dietary quality according to DASH and DII have been linked to cardiometabolic health outcomes and related mortality [11,13,14,15], conflicting results have emerged regarding both constructs [16,17,18]. In particular, recent compilations of studies have highlighted inconsistencies regarding the effect of dietary patterns on different biomarkers of systemic inflammation [16], where links between dietary quality and systemic inflammation were established in some but not all studies [19,20,21,22,23]. Furthermore, associations between dietary quality and adiponectin level, a major anti-inflammatory adipokine underlying several metabolic abnormalities, were attenuated after adjustment for adiposity [8]. Thus, it is imperative to consider body composition when exploring the true impact of dietary qualities on systemic inflammation. Furthermore, the confounding influence by variations in time spent in physical activity (PA) often relies on self-report, which is more prone to misclassification of physical activity compared to objective measurements. Finally, there is a paucity of studies specifically targeting older adults, which is unfortunate given the marked increase in metabolic risk factors [24] and elevated inflammatory status [25] associated with advancing age. Here, we hypothesize that dietary patterns are linked to inflammatory and metabolic biomarkers and that these links are independent of PA. Therefore, the aim of the present study is to determine links between two established dietary constructs and metabolic risk factors, encompassing pro- and anti-inflammatory biomarkers in older women, while considering objectively assessed time in physical activity.

## 2. Materials and Methods 

### 2.1. Subjects

A total of 122 community-dwelling women, between 65 and 70 years old, were recruited through local advertisement. To be included in the study, participants had to be free of diagnosed coronary heart disease and diabetes mellitus, have no disability with respect to mobility, not be using prescribed anti-inflammatory medication, and be non-smokers. Five women had incomplete data on PA and five had incomplete data on inflammatory and metabolic biomarkers. A total of 112 women fulfilled inclusion criteria. All procedures were conducted according to standards set by the Declaration of Helsinki. Written informed consent was obtained from all participants and the study was approved by the regional ethics committee of Uppsala (2011/033).

### 2.2. Assessment of Inflammatory and Metabolic Risk Factors

Blood samples collected by venipuncture were obtained after an overnight fast. Fibrinogen and high-sensitivity C-reactive protein (Hs-CRP) were assessed using an automated immunoassay method with a polyclonal rabbit anti-human antibody (Dako, Glostrup, Denmark) and a fully automated immunoturbidimetric assay (Advia 1800, Chemistry System, Siemens, Germany), respectively. Adiponectin was assessed using a commercially available ELISA kit (Mercodia, Uppsala, Sweden). Fasting blood triglycerides and high-density lipoprotein (HDL)-cholesterol were assessed using chemistry kits from Ortho-Clinical Diagnostics on a Vitros-5.1 analyser platform (Clinical Diagnostics, Raritan, NJ, USA). Blood glucose was assessed using the Reflotron Plus^®^ system (Roche Diagnostics Limited, Rotkreuz, Switzerland). Systolic and diastolic blood pressure were measured manually after a 15-min rest in the supine position using a mercury sphygmomanometer. Waist circumference was measured with a steel tape at the midpoint between the iliac crest and lower costal margin. A clustered metabolic risk score based on five established markers of metabolic risk (levels of triglycerides, HDL-cholesterol, blood glucose, waist circumference and mean blood pressure) was created, as previously described [26]. In brief, each outcome variable was standardized (*z*-scores) and the average score out of the five standardized variables was calculated. 

### 2.3. Assessment of Dietary Quality

Dietary intake was assessed using a 6-day food record. Participants were instructed by a nutritionist on how to register daily food intake with the assistance of a portion size guide developed by the Swedish National Food Agency. The dietary intake, including total energy, was analysed using Dietist XP software (Kost och Näringsdata, Bromma, Sweden). The DII score was derived from 30 food items following calculation procedures previously presented [12]. In brief, by using global daily mean and standard deviations (SDs), percentiles were obtained for each food item and multiplied with its corresponding inflammatory effects score before being summed together to obtain the overall DII score. 

The DASH-style diet was assessed according to standards set by Fung et al. [27], where participants’ intakes from eight food items/groups divided into favourable (fruits, vegetables, nuts and legumes, whole grains, low-fat dairy) and unfavourable (red and processed meats, sweetened beverages, sodium) food are ranked by quintiles. Hence, each quintile represents a score ranging from 1–5, with a low score representing a low intake and vice versa. The scores for each food component were summed up to produce an overall DASH score in the range of 8–40, with a higher score representing higher adherence to the DASH-style diet. 

### 2.4. Assessment of Physical Activity (PA)

Accelerometer-based assessment of PA during a week was performed using a waist-mounted Actigraph GT3x activity monitor (Actigraph, Pensacola, FL, USA), as previously described [26]. Briefly, the monitor was worn on awake time with a minimum of 4 days and 600 min per day necessary for inclusion. Time in moderate-to-vigorous PA (MVPA) was assessed based on a cut-point of ≥2020 counts per min in accordance with previous work [28] and adjusted by wear time prior to analysis.

### 2.5. Statistical Analyses

All data are presented as means ± standard deviation (SD) unless otherwise noted. Skewed data were log-transformed prior to analysis. First, relationships between dietary constructs (DII and DASH scores) and metabolic and inflammatory outcomes were investigated by Spearman rank order correlations. Second, analysis of covariance (ANCOVA) was employed to investigate differences in metabolic and inflammatory outcomes across tertiles of DII and DASH adherence. Notably, the DASH tertiles were not mathematically derived, as otherwise participants sharing the same DASH score would have been placed in different groups. Main effects of DII and DASH adherence were assessed in separate models. Each model was adjusted by use of prescribed medication as a binary outcome (yes/no) and total energy intake and time spent in MVPA as continuous covariates. All analyses were additionally adjusted by waist circumference, except when analysing the clustered metabolic risk score. In cases where significant main effects were observed, a post-hoc analysis with Bonferroni correction was conducted to determine effects between tertiles. A priori power calculation revealed that moderate effect sizes (≥0.4) would be detected with a power of ≥80% when performing all ANCOVA models with alpha (α) set to *p* < 0.05. All analyses were performed using SPSS 25.0 software (SPSS, Chicago, IL, USA).

## 3. Results

The final sample of 112 women had a mean height of 165 ± 5.8 cm, mean weight of 69 ± 11.5 kg and body mass index of 25.4 ± 4.1 kg/m^2^. Subject characteristics are shown in Table 1. Daily intakes of DASH-related food groups for the whole study sample are presented in Table S1 (see supplementary materials). 

The average DII score was 0.47 ± 1.73 for the whole sample and −1.37 ± 1.0, 0.51 ± 0.45 and 2.35 ± 0.78 for tertiles 1, 2 and 3, respectively. The median (range) DASH-score was 24 (12 to 34) for the whole sample and 119 (12–22), 24 (23–26) and 29 (27–34) for tertiles 1, 2 and 3, respectively. 

As expected, the two dietary constructs were inversely related to each other (rs = −0.4; *p* < 0.01). The average wear time of the activity monitor was 14.2 ± 1.0 h on 5.8 ± 0.5 days. 

Variables of metabolic risk and inflammatory biomarkers for the whole study sample and by tertiles of DASH adherence and DII are shown in Table 2 and Table 3, respectively. Based on established criteria by the International Diabetes Federation, 48 women (43%) had alterations in metabolic risk factors defined as metabolic syndrome.

Significant inverse relationships were observed between the DASH-score and both waist circumference (rs = −0.34; *p* < 0.01) and blood glucose level (rs = −0.32; *p* < 0.01). Further, while the DASH-score was significantly related to the anti-inflammatory biomarker adiponectin (rs = 0.29; *p* < 0.01), no corresponding associations to the pro-inflammatory biomarkers fibrinogen and CRP were observed. There were no significant associations between the DII score and any metabolic or inflammatory factor.

**Figure 1:**
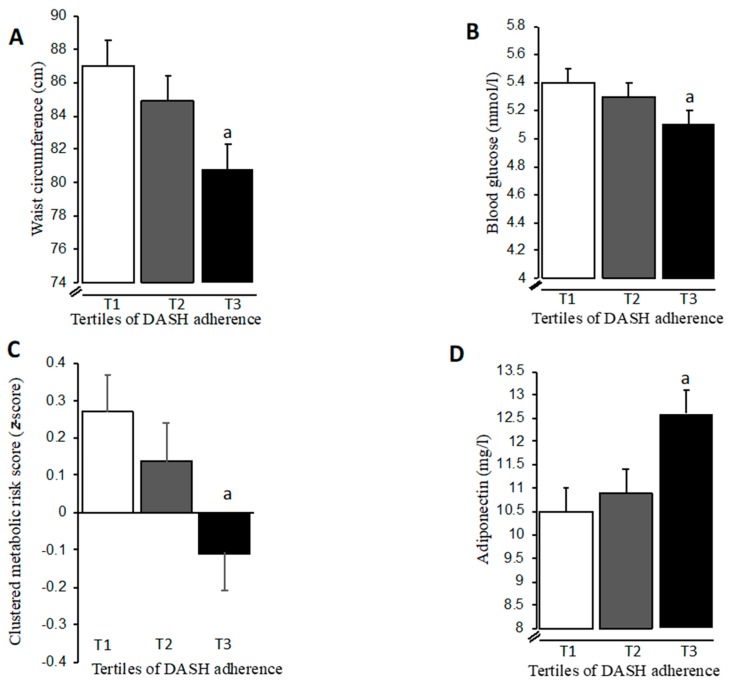
Adjusted means (±SEM) of waist circumference (**A**), blood glucose (**B**), clustered metabolic risk (**C**) and adiponectin level (**D**) across tertiles (T1, T2, T3) of DASH adherence in older women. ^a^ *p* < 0.05 compared to T1. Means are adjusted by total energy intake, medication and time in MVPA, and additionally by waist circumference (blood glucose and adiponectin).

When investigating effects on metabolic and inflammatory factors across tertiles of DASH adherence, significant main effects (*p* < 0.05) on waist circumference and blood glucose level were evident (Table 2). After further adjustment by total energy intake, medication and time in MVPA, women belonging to the highest DASH adherence group had 7% smaller (*p* < 0.05) waist circumferences and 6% lower (*p* < 0.05) blood glucose levels compared to those in the lowest DASH adherence group (Figure 1A,B). Notably, the between-tertile difference in blood glucose level was controlled for waist circumference (Figure 1B). When expressing metabolic risk as a clustered composite score, significant main effects across tertiles of DASH adherence were also observed (Table 2), where those belonging to the highest DASH adherence group had a more favourable risk score after adjustment for covariates (Figure 1C). Importantly, a significant main effect (*p* < 0.05) on adiponectin level across DASH tertiles was observed (Table 2) that remained after adjustment by total energy intake, medication, time in MVPA and waist circumference. Women in the highest DASH adherence group had 20% higher (*p* < 0.05) levels of adiponectin compared to those in the lowest adherence group (Figure 1D). Finally, no effects on metabolic or inflammatory factors were observed across tertiles of DII (Table 3).

## 4. Discussion

The current study provides novel insights into how dietary quality relates to important metabolic and inflammatory risk factors in older women. High adherence to the DASH-style diet promotes an anti-inflammatory systemic environment through elevated adiponectin levels, accompanied by a favourable clustered metabolic risk profile, even when variations in abdominal obesity are considered. A key finding is that objectively assessed time in MVPA does not attenuate the DASH-related health outcomes in older women, which further strengthens healthy eating behaviours as a key target for clinical and public health interventions designed to prevent age-related metabolic abnormalities.

The present study provides evidence for a DASH-related modulation of the systemic inflammatory profile in older women, where a high adherence to DASH-style diet favours elevated levels of the anti-inflammatory adiponectin. Being an adipocyte-derived adipokine, the release of adiponectin is closely related to adipose tissue function. Indeed, a robust association between excess adipose tissue and reduced adiponectin levels has previously been established [29]. An additional mechanism underpinning reduced adiponectin secretion is an adipocyte dysfunction related to insulin resistance [29]. An important finding in our study is that variations in waist circumference did not alter the link between high adherence to DASH-style diet and adiponectin. Thus, the diet-related differences in adiponectin levels in our study may not only be mediated by variations in total fat mass but also through an added effect on the regulation of adipocyte function. Another important finding in the present study is that diet-related links to adiponectin are independent of variations in MVPA time. Interestingly, we previously reported no links between PA level and adiponectin in older women, though waist circumference was related to MVPA time [6,26]. Thus, healthy eating patterns may alleviate adipocyte dysfunction and promote elevated adiponectin levels through pathways independent of physical activity. This finding provides support to earlier reports suggesting the existence of links between healthy diet and adiponectin levels in women [8,30,31].

In contrast to its favourable link to the anti-inflammatory biomarker adiponectin, adherence to DASH-style diet was not significantly associated to variations in circulating levels of the pro-inflammatory markers CRP and fibrinogen. In fact, links between DASH-style diet and levels of both CRP and fibrinogen have been observed in some [20,32] but not all studies [22,33]. Moreover, it is suggested that the impact of diet quality on circulating CRP level in experimental settings may in fact be mediated through a diet-induced weight loss [16], and a low overall strength of evidence on links between DASH and CRP has recently been concluded due to study inconsistencies and imprecision in effect estimates [34]. Interestingly, we have previously shown that variations in circulating CRP and fibrinogen levels in older women are sensitive to different PA intensities [6]. Thus, it may be hypothesized that PA behaviours have a stronger influence on CRP and fibrinogen than dietary patterns, and that the mechanisms underpinning the PA-related influence on these pro-inflammatory markers are independent of those induced by diet quality. 

Our study shows that adherence to DASH-style diet is linked to a more favourable clustered metabolic risk profile, where women in the highest DASH tertile had a smaller waist circumference and a lower blood glucose level compared to women in the lowest tertile. While healthy dietary patterns are linked to body composition [35], associations to blood glucose are less clear [11,34]. Nonetheless, our findings suggest that adherence to DASH-style diet improves clustered metabolic risk profile in older women through its influence on both abdominal obesity and blood glucose levels.

Of note, no association between DASH-style diet and blood pressure was observed in our sample. This may seem surprising given that DASH was originally developed for the non-pharmacological treatment of hypertension. However, a compilation of studies noted that while DASH can infer a clinically relevant reduction in blood pressure [36], this effect was largely influenced by a concomitant energy restriction and was particularly observed in studies with hypertensive participants.

While the DASH and DII scores were significantly related to each other, the DII was not associated with any inflammatory or metabolic risk outcome. Interestingly, the lack of associations with metabolic risk outcomes confirms previous reports based on larger study populations [37,38]. Furthermore, in a study based on more than 2500 middle-aged men and women, no links between DII and CRP or fibrinogen could be established [19], suggesting that dietary patterns in general, with the DII in particular, cannot reliably predict variations in levels of these two clinically important biomarkers of inflammation. However, it should be noted that the systemic inflammatory status is complex, involving a large array of inter-related biomarkers originating from different tissues. Hence, even though no links to CRP and fibrinogen were observed in the present study, several other biomarkers may be susceptible to being influenced by different aspects of dietary quality defined by the DII score. The fact that the DII has been related to inflammatory biomarkers [21] and to risk of inflammation-induced atherosclerosis in older women [14] suggests that the DII is able to at least partly capture dietary qualities relevant to predicting health outcomes, even though it may not be as robust as the DASH construct.

Some limitations must be considered while interpreting our findings. As in any cross-sectional study, causation cannot be inferred and findings may not be generalizable to broader populations of older men and women with varying health status. In our study, the DII was derived from 30 of the original 45 food items. However, DII scores in several previous studies were based on equal or fewer food items [19,21,39].

In conclusion, DASH-style diet promotes an anti-inflammatory systemic environment through elevated adiponectin level and a favourable clustered metabolic risk profile, regardless of variations in abdominal obesity. Importantly, DASH-related health outcomes are further independent of MVPA time, which strengthens healthy eating behaviours as a key target for clinical and public health efforts aiming to combat age-related metabolic abnormalities.

## Figures and Tables

**Table 1:** Subject characteristics in the study sample and across tertiles of DASH-adherence.

Subject Characteristics	Tertile 1	Tertile 2	Tertile 3	Total
Age (year)	67.5 (1.7)	67.3 (1.5)	67.5 (1.7)	67 (1.6)
Total energy intake (Kcal)	1675 (332)	1689 (372)	1706 (427)	1689 (372)
Daily time in MVPA (min)	23 (16)	31 (23)	38 (32)	30 (24)
Medication (% yes)	32	31	34	32

Data are mean (SD) unless indicated. DASH: Dietary Approach to Stop Hypertension; MVPA: moderate-to-vigorous physical activity.

**Table 2:** Inflammatory and metabolic biomarkers in the study sample and across tertiles of DASH-adherence.

Risk Outcomes	Total	Tertile 1	Tertile 2	Tertile 3	*p*-Value
Waist circumference (cm)	84 (11)	88 (9)	84 (11)	79 (11) ^a^	*p* = 0.002
Triglycerides (mmol/L)	1.2 (0.4)	1.2 (0.5)	1.1 (0.4)	1 (0.3)	*p* = 0.21
HDL-cholesterol (mmol/L)	1.6 (0.4)	1.5 (0.3)	1.5 (0.3)	1.7 (0.4)	*p* = 0.12
Glucose (mmol/L)	5.3 (0.6)	5.5 (0.6)	5.3 (0.5)	5 (0.7) ^a^	*p* = 0.005
Systolic blood pressure (mmHg)	136 (15)	134 (15)	134 (16)	139 (14)	*p* = 0.32
Diastolic blood pressure (mmHg)	78 (8)	79 (9)	76 (9)	79 (7)	*p* = 0.24
CRP (mg/L) *	1.1 (1.5)	1.3 (1.6)	1 (1.7)	1 (0.8)	*p* = 0.26
Fibrinogen (g/L)	3.2 (0.6)	3.2 (0.5)	3.2 (0.7)	3.3 (0.6)	*p* = 0.64
Adiponectin (mg/L)	11.5 (3.4)	10.6 (3.4)	11.2 (3.3)	12.9 (3.3) ^a^	*p* = 0.008

Data are means (SD) unless indicated. * Geometric mean. *p*-values denote ANOVA main effects across tertiles. ^a^ denotes significant difference between T1 and T3. CRP: C-reactive protein.

**Table 3:** Inflammatory and metabolic biomarkers across tertiles of the dietary inflammatory index (DII).

Risk Outcomes	Tertile 1	Tertile 2	Tertile 3	*p*-Value
Waist circumference (cm)	84 (12)	82 (11)	86 (9)	*p* = 0.39
Triglycerides (mmol/L)	1.1 (0.4)	1.1 (0.4)	1.2 (0.5)	*p* = 0.79
HDL-cholesterol (mmol/L)	1.6 (0.4)	1.5 (0.3)	1.6 (0.4)	*p* = 0.62
Glucose (mmol/L)	5.3 (0.7)	5.2 (0.5)	5.3 (0.5)	*p* = 0.95
Systolic blood pressure (mmHg)	136 (15)	138 (15)	133 (15)	*p* = 0.26
Diastolic blood pressure (mmHg)	77 (7)	78 (10)	77 (8)	*p* = 0.95
CRP (mg/L) *	1 (1.8)	1.1 (1.3)	1.2 (1.2)	*p* = 0.65
Fibrinogen (g/L)	3.2 (0.6)	3.3 (0.5)	3.2 (0.8)	*p* = 0.72
Adiponectin (mg/L)	11.3 (3.6)	11.4 (3.2)	11.7 (3.4)	*p* = 0.88

Data are means (SD) unless indicated. * Geometric mean.

## Supplementary Materials

The following are available online at https://www.mdpi.com/2072-6643/11/4/805/s1, Table S1. Daily intake of DASH-related food groups in the study sample.
