# Supplementary material for: Adherence to DASH-Style Dietary Pattern Impacts on Adiponectin and Clustered Metabolic Risk in Older Women
Source: Nutrients. 2019 Apr 9;11(4):805. doi: 10.3390/nu11040805 (PMC6520850; doi:10.3390/nu11040805)
Supplement: Supplementary file 1 [file nutrients-11-00805-s001.pdf]

Table S1. Daily intake of DASH-related food groups in the study sample. DASH: Dietary Approach to Stop Hypertension.

**Food groups**	**Median (interquartile range)**
Fruits (g)	249 (151–352)
Vegetables (g)	135 (98–196)
Nuts and legumes (g)	6.5 (0–25)
Whole grains (g)	31 (20–48)
Low fat dairy (g)	0 (0–150)
Red and processed meats (g)	83 (56–107)
Sweetened beverages (g)	0 (0–42)
Sodium (mg)	2250 (1860–2720)
